# Carnosic Acid Suppresses the Development of Oral Squamous Cell Carcinoma *via* Mitochondrial-Mediated Apoptosis

**DOI:** 10.3389/fonc.2021.760861

**Published:** 2021-11-26

**Authors:** Fenghe Min, Xin Liu, Yuan Li, Mingyuan Dong, Yidi Qu, Weiwei Liu

**Affiliations:** ^1^ Department of Oral and Maxillofacial Surgery, Hospital of Stomatology, Jilin University, Changchun, China; ^2^ Jilin Provincial Key Laboratory of Tooth Development and Bone Remodeling, Changchun, China; ^3^ School of Life Sciences, Jilin University, Changchun, China

**Keywords:** carnosic acid, oral squamous cell carcinoma, reactive oxygen species, mitochondria, apoptosis

## Abstract

Oral squamous cell carcinoma (OSCC) predominantly consists of squamous cells and is the tumor with the highest incidence of the head and neck. Carnosic acid (CA), a natural monomer drug obtained from rosemary and salvia, shows various pharmacological effects, including of tumor development. This study aimed to assess for an effect of CA on the development of OSCC and the underlying mechanisms. In CAL27 and SCC9 cells, CA inhibited cell proliferation and migration, increased intracellular levels of reactive oxygen species (ROS) and Ca^2+^, decreased the mitochondrial membrane potential (MMP), and promoted apoptosis. In CAL27- and SCC9-xenotransplanted BALB/c nude mice, CA inhibited the tumor growth without affecting the body weight and tissue morphology. CA upregulated Bax, Bad, cleaved Caspase-3 and -9 levels, and the cleaved PARP1/PARP1 ratio but downregulated Bcl-2 in CA-treated OSCC cells and OSCC cells-xenotransplanted BALB/c nude mice. These results indicate that CA suppresses OSCC at least *via* the mitochondrial apoptotic pathway and offers this natural compound as a potential therapeutic against OSCC.

## Introduction

Oral cancer is any malignant tumor caused by abnormal changes in the oral mucosa. The cases are predominantly linked to oral squamous cell carcinoma (OSCC) and rarely to salivary-gland cancers (1, 2). OSCC is a typical head and neck cancer, accounting for 2.8% of all the cancers in the world (3). Due to its high incidence, > 200,000 die from OSCC worldwide every year (3). Most patients have an unfavorable prognosis due to the anatomical location of this cancer, lymph node metastasis, or recurrence (4, 5). Smoking, drinking, and betel nut chewing are considered to be responsible for the development of OSCC (6, 7).

Apoptosis is a form of programmed cell death, mediated by multiple genes and cytokines, and constitutes an important target pathway to kill tumor cells (8, 9). Cellular stress, DNA damage, and developmental signals all activate the endogenous apoptotic pathway (9–11). Mitochondria are the primary source of intracellular reactive oxygen species (ROS) (12), which are oxygen-containing free radicals. ROS affect the influx of Ca^2+^ into cells and cellular calcium stores, and Ca^2+^ increases the production of ROS (13). The accumulation of ROS and Ca^2+^ regulates the access through the mitochondrial permeability transition pore (mPTP) and decreases the potential while increasing the permeability of the mitochondrial membrane, whereby cytochrome c (Cyt-c) is released into the cytoplasm and activate the mitochondrial apoptotic pathway (14–16). B-cell lymphoma 2 (Bcl-2) family proteins are involved in this process. When the permeability of the mitochondrial membrane increases, apoptotic bodies translocate from the mitochondria to the cytoplasm and consequently activate caspase-family proteins, causing DNA damage and ultimately leading to apoptosis (17–19).

Currently, OSCC is treated *via* surgery, radiotherapy, and chemotherapy (20, 21). However, surgery is only applicable to early lesions and has a poor prognosis. Radiotherapy and chemotherapy may cause osteoradionecrosis (ORN), myelosuppression, and organ damage, significantly affecting the life qualities of patients (22). Thus, novel drugs with high efficacy and low toxicity are urgently needed. Recently, natural compounds have increasingly attracted attention and have been widely used to develop anticancer drugs (23). Carnosic acid (CA), a phenolic diterpene compound, is mainly found in labyrinthine plants and has been shown to have various pharmacological effects, such as antioxidant, antitumor, anti-inflammatory, and neuroprotective effects (24–27). CA induced apoptosis in hepatocellular carcinoma cells through the ROS-mediated mitochondrial pathway (28). It has also been shown to induce apoptosis in human gastric cancer cells by activating the protein kinase B/mammalian target of rapamycin (Akt/mTOR) signaling pathway (29). CA induced apoptosis of HCT116 cells by inhibiting the STAT3 signaling pathway (30). Moreover, CA synergized the anti-lung cancer effect of cisplatin and the anti-breast cancer effect of Trastuzumab (31–33). However, there are no relevant reports on the effect of CA in OSCC. Thus, this study assessed whether CA can suppress the development of OSCC.

## Materials and Methods

### Cell Culture

CAL27 (CRL-2095) and SCC9 (CRL-1629) cells (human OSCC cell lines) were obtained from the American Type Culture Collection (ATCC) and cultured in Dulbecco’s Modified Eagle Medium (DMEM) (Gibco, Grand Island, New York, USA) and DMEM/F12 (Hyclone, Logan, Utah, USA) at 37°C with 5% CO_2_, respectively. The two media contained 10% fetal bovine serum (FBS) (Procell Life Science&Technology Co., Ltd., Wuhan, Hubei, China), 1% penicillin and streptomycin (Sigma-Aldrich, Saint Louis, Missouri, USA), and 0.1% plasmocin prophylactic (*In vivo*gen, San Diego, California, USA).

### Cell Viability Assay

CAL27 and SCC9 cells at the logarithmic growth phase were plated into 96-well plates at a density of 8 × 10^3^ cells per well and cultured for 24 h. Then, the cells were incubated with 0, 5, 10, 20, 30, 40, 60, or 80 μM CA (B21175, Shanghai Yuanye Biological Technology Co., Ltd., Shanghai, China), which had been dissolved in dimethyl sulfoxide (DMSO) (Sinopharm Chemical Reagent Co., Ltd., Shanghai, China). The final concentration of DMSO in the culture was ≤ 0.1%. After 24 h of CA treatment, the cells in each well were incubated with 10 μL of 5 mg/mL 3-(4,5-dimethylthiazolyl-2)-2,5-diphenyltetrazolium bromide (MTT) (S19063, Source Leaf Biological Technology Co., Ltd., Shanghai, China) for 4 h. Afterward, the culture supernatant was discarded, and 150 μL DMSO was used to dissolve the formazan crystals in each well. Absorbance of the samples was measured at 490 nm using an Enzyme-labeled Instrument (HBS-1096A, NanJing DeTie Laboratory Equipment Co., Ltd., Nanjing, Jiangsu, China).

### Migration Assay

CAL27 and SCC9 cells at the logarithmic growth phase were plated into 6-well plates at a density of 3 × 10^5^ cells per well. When the cells reached > 90% confluence, they were scraped using a syringe needle and then cultured with CA (30 and 15 μM for CAL27 and SCC29 cells, respectively) for 0, 12 and 24 h. Fluorescence microscope (Eclipse TE 2000-S, Nikon Corp., Tokyo, Japan) and quantifiable ImageJ software were used to analyze the influence of CA on OSCC cells migration.

### Apoptosis Assay

CAL27 and SCC9 cells at the logarithmic growth phase were plated into 6-well plates at a density of 2.5 × 10^5^ cells per well and incubated in an incubator at 37°C with 5% CO_2_ for 24 h. Next, the CAL27 and SCC9 cells were incubated with 30 and 15 μM CA for 12 h, respectively. Afterward, the cells were processed using the eBioscietm Annexin V-FITC Apop Kit (BMS500FI-100, Invitrogen, Carlsbad, California, USA) according to the instructions of the manufacturer and analyzed using a CytoFLEX Flow Cytometer (C02945, Beckman Coulter, Inc. Brea, Carlsbad, California, USA).

### Mitochondrial Membrane Potential (MMP) Assay

CAL27 and SCC9 cells at the logarithmic growth phase were plated into 6-well plates at a density of 2.5 × 10^5^ cells per well and cultured for 24 h. Subsequently, the CAL27 and SCC9 cells were incubated with 30 and 15 μM CA for 12 h, respectively. Afterward, the cells were washed with phosphate-buffered saline (PBS) three times and then incubated with 5 mg/mL 5,5′,6,6′-Tetrachloro-1,1′,3,3′-tetraethyl-benzimidazolylcarbocyanine iodide (JC-1) (C2006, Beyotime Biotechnology, Shanghai, China) for 20 min. Fluorescence intensity of the samples was measured using a fluorescence microscope (Eclipse TE 2000-S, Nikon Corp., Tokyo, Japan), and the related data were quantitatively analyzed using ImageJ software.

### Quantitation of Intracellular ROS and Ca^2+^

CAL27 and SCC9 cells at the logarithmic growth phase were plated into 6-well plates at a density of 2.5 × 10^5^ cells per well and then incubated for 24 h. Afterward, the CAL27 and SCC9 cells were exposed to 30 and 15 μM CA for 12 h, respectively. The cells were incubated with 10 μM DFCH-DA for 30 min, and the intracellular ROS levels were measured using the Reactive Oxygen Species (ROS) Assay Kit (S0033S, Beyotime Biotechnology, Shanghai, China) according to the instructions of the manufacturer.

For Ca^2+^ detection, the cells were incubated with 1 μM Fluo-4AM (S1060, Beyotime Biotechnology, Shanghai, China) for 40 min. Afterward, the culture supernatant was discarded, and the cells were washed three times with PBS. Fluorescence intensity of the samples was measured using a fluorescence microscope (Eclipse TE 2000-S, Nikon Corp., Tokyo, Japan), and the related data were quantitatively analyzed using ImageJ software.

### Transmission Electron Microscopy (TEM) Analysis

The incubation and CA treatment protocols of CAL27 and SCC9 cells were the same as *2.6 Quantitation of intracellular ROS and Ca^2+^*.The collected cells were fixed at 4°C for 4 h, embedded in 1% agarose solution, and then fixed in 1% osmium tetroxide for the secondary fixation. After dehydration with ethanol, the cells were embedded in the embedding plate and polymerized for 48 h. The ultra-microtome (Leica UC7, Leica, Weztlar, Germany) was used to cut the resin blocks into 60-80 nm and loaded the slices on a 150-mesh cuprum grids. After the sections were stained with uranyl acetate and lead citrate, images were captured using a transmission electron microscope (H-7650, HITACHI, Japan).

### CAL27- and SCC9-Xenograft Models

All the animal experiments were conducted under the guidance of the Animal Ethics and Welfare Committee of Jilin University (NO. SY202101004). Male BALB/c nude mice (5 weeks old) (Wei-tongli-hua Laboratory Animal Technology Company, Beijing, China) were provided with sufficient food and water and maintained at 23 ± 1°C under 12 h/12 h light/dark cycle.

CAL27 and SCC9 cells (1 × 10^7^) were subcutaneously inoculated into the right dorsum of BALB/c nude mice. When the tumor volume reached 100 mm^3^, the mice in each group were randomly divided into two sub-groups (n = 6 per sub-group) and intraperitoneally injected every other day for 14 d with the physiological saline containing < 0.1% DMSO (control mice) or 20 mg/kg of CA dissolved in DMSO with a final concentration of < 0.1% (CA-treated mice). The volumes of the tumors and the body weights of the mice were measured before each administration. Tumor volume was calculated according to the following formula: length (mm) × [width (mm)]^2^ × 0.5. After the last treatment, the mice were euthanized *via* carbon-dioxide asphyxiation. The tumors were removed and homogenized for western blot analysis. Peripheral-blood samples were collected from the mice for blood analyses using an automatic blood analyzer. The tumor, heart, liver, spleen, and kidney of the mice were fixed in 4% polyformaldehyde (POM) (AR0211, Dingguo Changsheng Biotechnology Co., Ltd., Beijing, China) for 48 h for histopathological examination.

### Histopathological Examination

Following the POM fixation, the samples were embedded in paraffin and then sectioned at 5 μm thickness by using a microtome (Leica, Wetzlar, Germany). The sections were stained with hematoxylin and eosin by using a standard protocol. After the slices were dehydrated and cleared with ethanol and xylene, images were captured using an Eclipse TE 2000-S fluorescence microscope (Nikon Corp., Tokyo, Japan).

### Terminal Deoxynucleotidyl Transferase-Mediated dUTP *In Situ* Nick End Labelling (TUNEL) Assay

The paraffin sections prepared from tumor tissues were deparaffinized and repaired with proteinase K. After breaking the membrane with 0.1% triton, terminal deoxynucleotidyl transferase (Tdt) enzyme and dUTP were added according to the instructions of TUNEL assay kit (G1205, Servicebio technology Co., Ltd., Wuhan, China), and incubated at 37°C for 2 h. The nuclei were stained with 2-(4-Amidinophenyl)-6-indolecarbamidine dihydrochloride (DAPI) and incubated in the dark for 10 minutes. Fluorescent images were captured using an Eclipse TE 2000-S fluorescence microscope (Nikon Corp., Tokyo, Japan).

### Western Blot Analysis

CAL27 and SCC9 cells at the logarithmic growth phase were plated into 6-well plates at a density of 2.5 × 10^5^ cells per well and cultured for 24 h. Afterward, the CAL27 and SCC9 cells were treated with 30 and 15 μM of CA for 24 h, respectively. The treated cells and the tumor tissues obtained from the BALB/c nude mice were homogenized using the RIPA Lysis Buffer (PC101, EpiZyme, Shanghai, China). The total-protein concentrations of the samples were measured using a Standard BCA Protein Assay Kit (23225, Thermo Fisher Scientific, Waltham, Massachusetts, USA) following the manufacturer’s instructions. Lysates with 30–40 μg total protein were electrophoresed using the One-Step PAGE Gel Fast Preparation Kit (PG213, EpiZyme, Shanghai, China) at 90–120 V and then transferred onto a PVDF membranes (Merck Millipore, Billerica, MA, USA) at 100 V for 2 h. The membranes were blocked with the NcmBlot blocking buffer (P30500, NCM Biotech, Suzhou, Jiangsu, China) and then incubated overnight at 4 °C with antibodies against Cleaved poly (ADP-Ribose) polymerase (PARP1) (A19612), PARP1 (A19596), Cleaved Caspase-3 (A2156), Caspase-3 (A2156), Caspase-9 (A2636), Bad (A19595), Bax (A19684) (all from ABclonal Technology Co., Ltd., Wuhan, Hubei, China); Cleaved Caspase-9 (Asp315, Cell Signaling Technology, Boston, Massachusetts, USA); B-cell lymphoma-2 (Bcl-2) (BSM-33047M, Beijing Biosynthesis Biotechnology Co., Ltd., Beijing, China); and Glyceraldehyde-3-phosphate dehydrogenase (GAPDH) (SY0102, Elabscience Biotechnology Co., Ltd., Wuhan, Hubei, China). After washing the membranes with TBST, they were incubated at 4°C for 4 h with the corresponding goat anti-rabbit IgG (H+L) (peroxidase/HRP-conjugated) (E-AB-1003) or anti-mouse IgG (H+L) (peroxidase/HRP-conjugated) (E-AB-1001) (Elabscience Biotechnology Co., Ltd., Wuhan, Hubei, China). The protein signals were detected using electrochemiluminescence (ECL) detection kits (Merck Millipore, Billerica, MA, USA), and the signal intensities were quantified using ImageJ software.

### Statistical Analysis

The statistical significance of the differences between the control and CA-treatment groups was determined using one-way analysis of variance (ANOVA). *Post-hoc* multiple comparisons (Dunn’s test) were performed using DSS 25.0 software (IBM Corporation, Armonk, New York, USA). *P* < 0.05 was considered significant.

## Results

### CA Induced Apoptosis in OSCC Cells *via* the Mitochondrial Pathway

**Figure 1:**
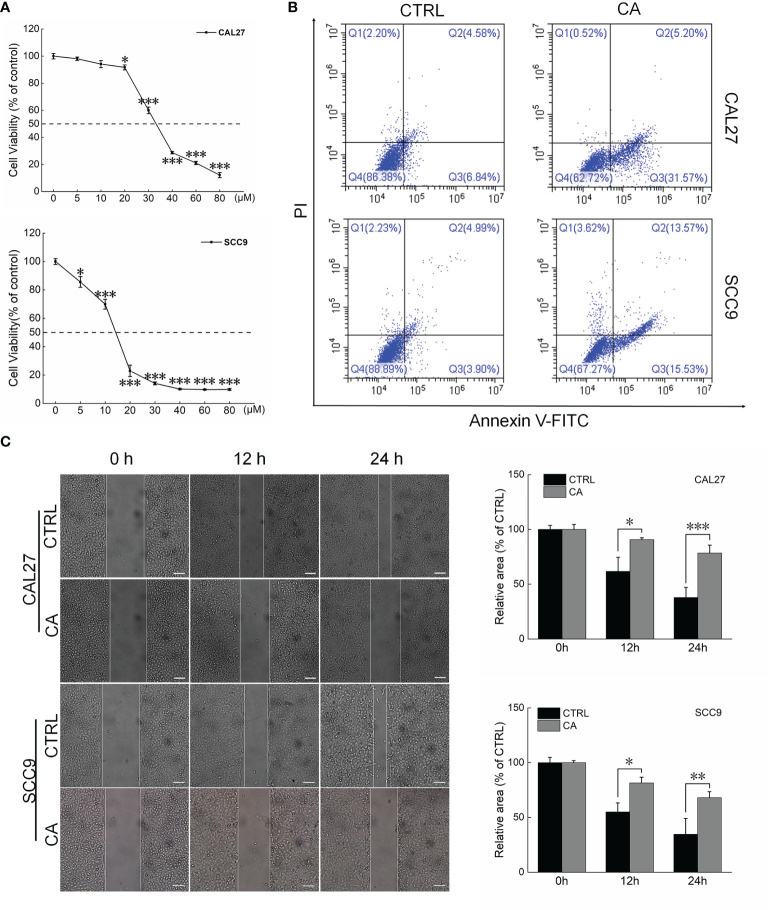
CA treatment affected the cell viability and promoted apoptosis in OSCC cells. **(A)** CA treatment for 24 h reduced the viability of CAL27 and SCC9 cells in a dose-dependent manner (*n* = 3). **(B)** CA treatment increased the apoptosis rates of CAL27 and SCC9 cells (*n* = 3). **(C)** CA treatment significantly inhibited the migration of CAL27 and SCC9 cells over time (*n* = 3) (200× magnification, scale bar: 50 μm). The data are expressed as mean ± SEM. *P < 0.05, **P < 0.01 and ***P < 0.001 *versus* the control cells.

CA significantly inhibited the viability of the OSCC cell lines CAL27 and SCC9 (IC_50_ 34.66 and 13.23 μM, respectively) (*P* < 0.05, **Figure 1A**). CA increased the early/late apoptosis of CAL27 and SCC9 cells to 36.77% and 29.1%, respectively (**Figure 1B**). The migration of OSCC cells was time-dependent. Compared with the control cells, CA treatment significantly inhibited the migration ability of these OSCC cells (*P <* 0.05, **Figure 1C**).

**Figure 2:**
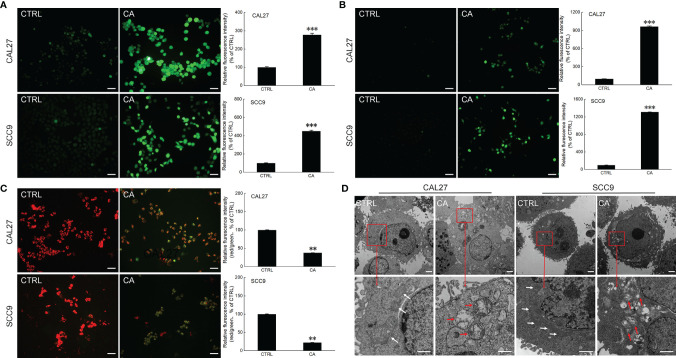
CA treatment affected the mitochondrial function in OSCC cells. CA treatment increased the intracellular **(A)** ROS and **(B)** Ca^2+^ levels and induced the **(C)** dissipation of the MMP in CAL27 and SCC9 cells (*n* = 3, 200× magnification, scale bar: 50 μm). **(D)** CA affected the structure of the mitochondria in OSCC cells observed by transmission electron microscope (TEM) (*n* = 3, 700× magnification, scale bar: 5.0 μm; 3000× magnification, scale bar: 1.0 μm). The quantitation results are expressed as percentages relative to the levels in the corresponding control cells, and the data are expressed as mean ± SEM. **P < 0.01 and ***P < 0.001 *versus* the control levels.

After CAL27 and SCC9 cells were incubated with CA for 12 h, increased green fluorescence intensity was observed, indicating that CA increased the ROS levels (*P* < 0.001, **Figure 2A**) and Ca^2+^ influx (*P* < 0.001, **Figure 2B**) in OSCC cells. Moreover, the measurement of mitochondrial membrane potential proved that after OSCC cells were exposed to CA, more green fluorescence from JC-1 monomer was observed, the intensity of the red fluorescence aggregated in the matrix of the mitochondria decreased, and the red/green fluorescence ratio was significantly reduced by > 60% in the CA-treated cells (*P* < 0.01, **Figure 2C**), indicating that the level of mitochondrial depolarization increased, and the MMP decreased, and thus the cells were in the early apoptotic stage. The images obtained by TEM showed that the mitochondrial cristae of CAL27 and SCC9 cells were arranged neatly and densely (**Figure 2D**). After CA incubation, the mitochondria were severely swollen, the matrix was dissolved, and the mitochondrial cristae was missing and accompanied by vacuoles (**Figure 2D**). The administration of CA affected the structure and function of mitochondria.

**Figure 3:**
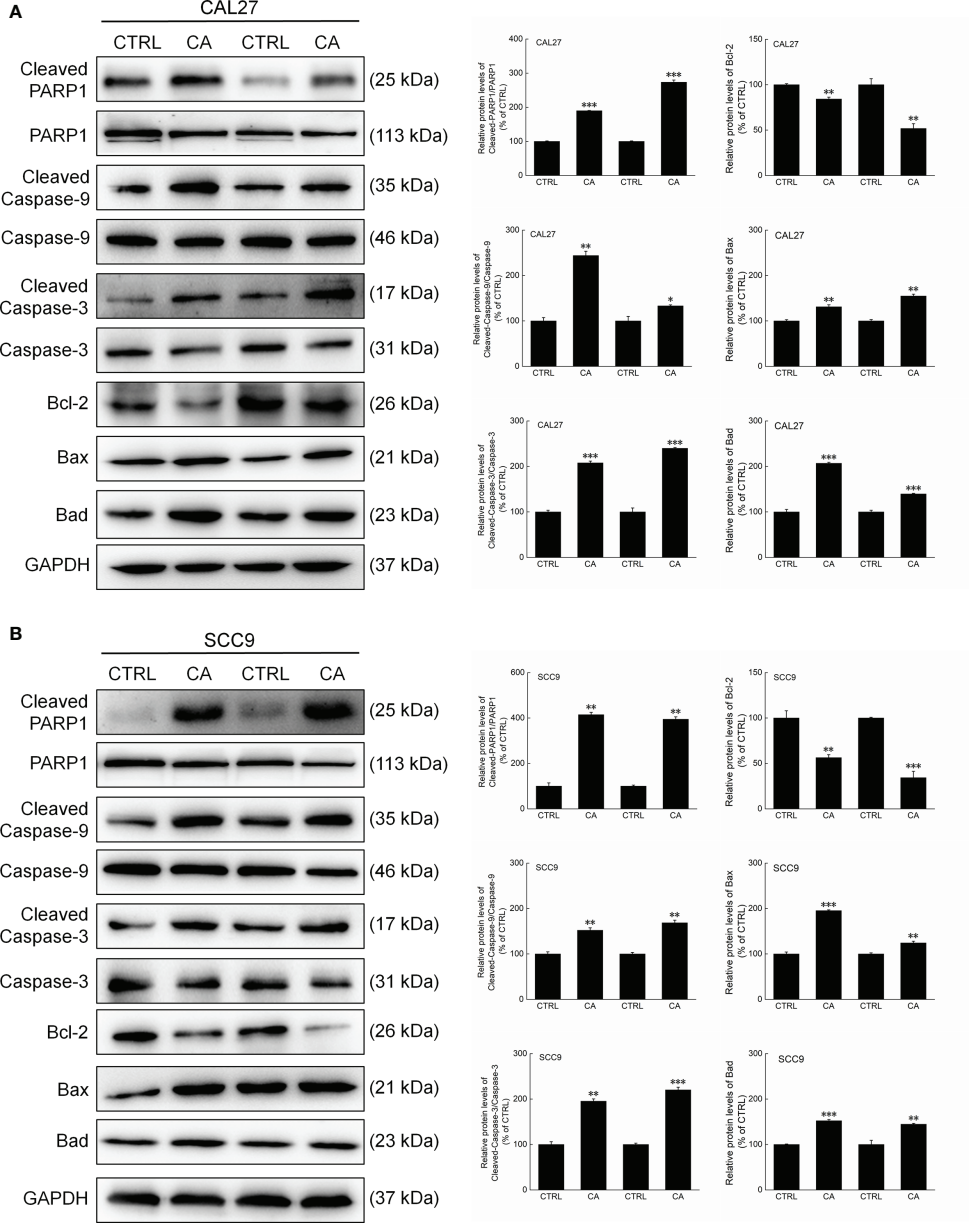
CA treatment changed the levels of apoptosis-related proteins in OSCC cells. CA treatment significantly enhanced the ratios of Cleaved PARP1/PARP1, Cleaved Caspase-3/Caspase-3, and Cleaved Caspase-9/Caspase-9; downregulated Bcl-2 and upregulated Bax and Bad in **(A)** CAL27 and **(B)** SCC9 cells (*n* = 3). The target-protein levels were normalized using the GAPDH levels, and the data are expressed as mean ± SEM. *P < 0.05, **P < 0.01 and ***P < 0.001 *versus* CTRL cells.

In CA-exposed CAL27 and SCC9 cells, the ratios of the levels of Cleaved PARP1, Caspase-3, and Caspase-9 to total PARP1, Caspase-3, and Caspase-9 levels, respectively, were increased (*P* < 0.05, **Figure 3**). Additionally, Bcl-2 was downregulated, whereas Bax and Bad were upregulated (*P* < 0.01, **Figure 3**). These *in vitro* results preliminarily showed that CA induced apoptosis in OSCC cells by reducing the MMP.

### CA Inhibited the Growth of CAL27- and SCC9-Xenotransplants by Inducing Their Apoptosis

**Figure 4:**
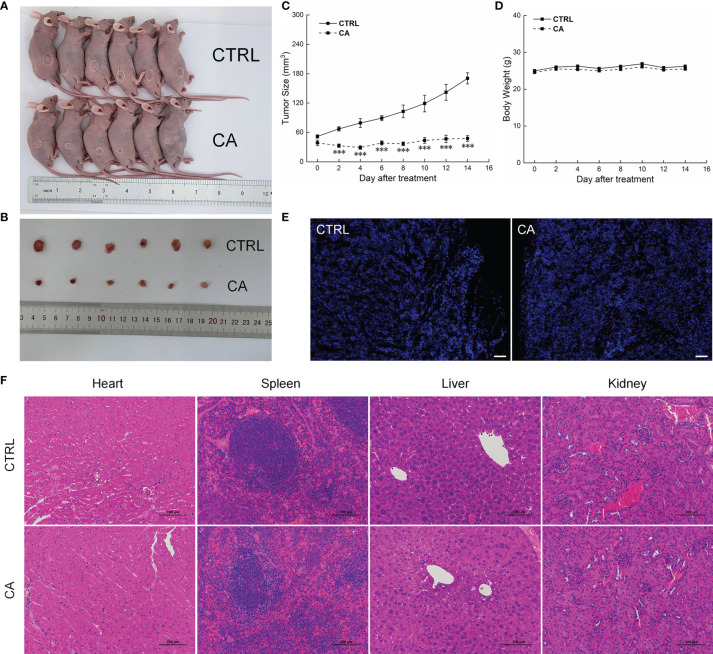
CA treatment inhibited the tumor growth in BALB/c nude mice transplanted with CAL27 cells. CA treatment significantly reduced tumor volumes in **(A)** tumor-bearing BALB/c nude mice and **(B)** tumor tissues collected from the control (CTRL) and CA-treated groups and **(C)** decelerated the tumor growth without affecting **(D)** the body weight of the mice (*n* = 6). **(E)** The apoptosis of tumor tissues increased after CA treatment (*n* = 3) (200× magnification, scale bar: 50 μm), while **(F)** the heart, spleen, liver, and kidney showed no histological abnormality (*n* = 3) (200× magnification, scale bar: 100 μm). Green fluorescence locates apoptotic cells. The data are expressed as mean ± SEM. ***P < 0.001 *versus* the CTRL group.

**Figure 5:**
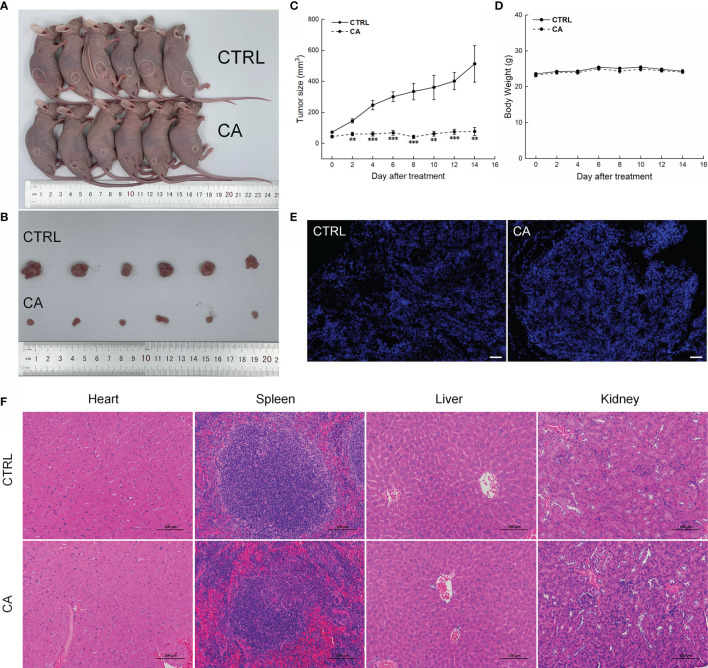
CA treatment inhibited the tumor growth in BALB/c nude mice transplanted with SCC9 cells. CA treatment significantly reduced tumor volumes in **(A)** tumor-bearing BALB/c nude mice and **(B)** tumor tissues collected from the control (CTRL) and CA-treated groups and **(C)** decelerated the tumor growth without affecting **(D)** the body weight of the mice (*n* = 6). **(E)** The apoptosis of tumor tissues increased after CA treatment (*n* = 3) (200× magnification, scale bar: 50 μm), while **(F)** the heart, spleen, liver, and kidney showed no histological abnormality (*n* = 3) (200× magnification, scale bar: 100 μm). Green fluorescence locates apoptotic cells. **P < 0.01 and ***P < 0.001 *versus* the CTRL group.

In CAL27- and SCC9-xenotransplanted BALB/c nude mice, CA treatment for 14 d significantly inhibited the tumor growth (*P* < 0.01, **Figures 4A**–**C** and **5A**–**C**) without affecting the body weights or organ indices of the animals (**Figures 4D** and **5D**; **Table S1**). Compared with CTRL mice, the green fluorescence in tumor tissues enhanced, in other words, the number of TUNEL positive cells increased after CA administration, indicating that CA administration promoted tumor tissue apoptosis (**Figures 4E** and **5E**). The cardiomyocytes and hepatocytes of the animals were still neatly arranged, the spleen had no significant infiltration of inflammatory cells, and the glomeruli had no obvious lesions, indicating that CA did not impact the histological features of the mice (**Figures 4F** and **5F**).

Results from the peripheral-blood analyses indicated increased monocyte number and decreased platelet distribution width (PDWcv) in the SCC9-xenotransplanted BALB/c nude mice (*P* < 0.05, **Tables S2**). No significant changes in other indicators were found in the mice transplanted with the OSCC cells (**Tables S2**).

**Figure 6:**
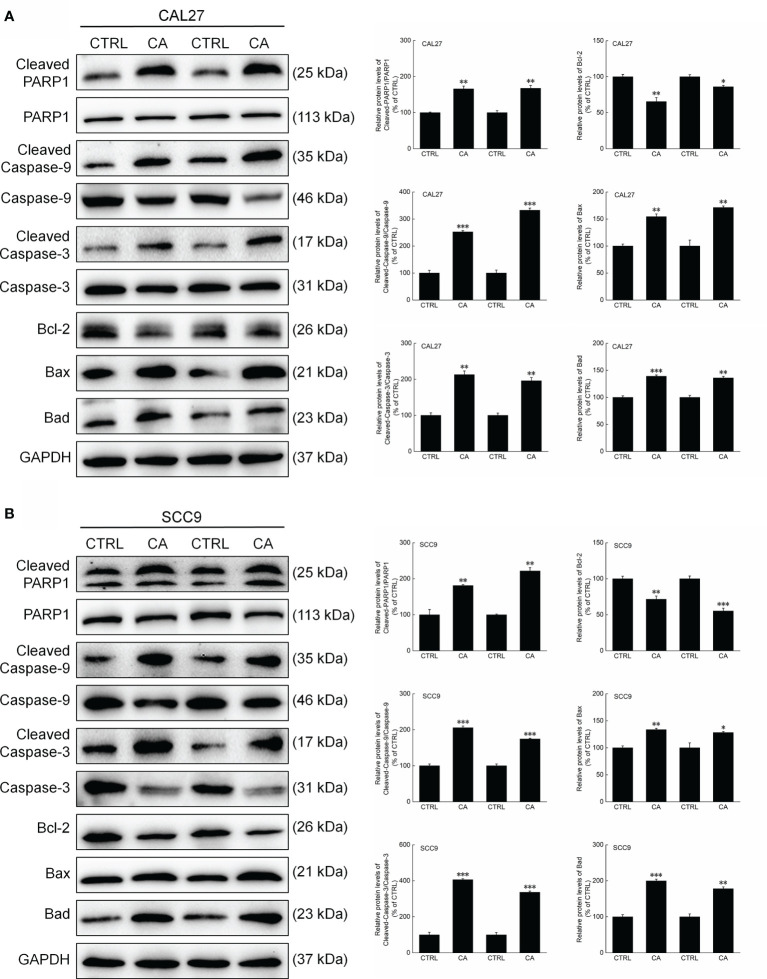
CA treatment changed the levels of apoptosis-related proteins in the tumor tissues collected from the BALB/c nude mice transplanted with OSCC cells. CA treatment significantly enhanced the ratios of Cleaved PARP1/PARP1, Cleaved Caspase-3/Caspase-3, and Cleaved Caspase-9/Caspase-9; downregulated Bcl-2 and upregulated Bax and Bad in the **(A)** CAL27- and **(B)** SCC9-xenografts in BALB/c nude mice (*n* = 3). The target-protein levels were normalized using the GAPDH levels, and the data are expressed as mean ± SEM. *P < 0.05, **P < 0.01 and ***P < 0.001 *versus* CTRL group.

Consistent with the *in vitro* results, CA administration for 14 d increased the ratios of the levels of Cleaved PARP1, Caspase-3, and Caspase-9 to total PARP1, Caspase-3, and Caspase-9 levels in the xenografts, respectively (*P* < 0.01, **Figure 6**). Furthermore, Bax and Bad were upregulated, whereas Bcl-2 was downregulated (*P* < 0.05, **Figure 6**).

## Discussion

The use of natural compounds against cancer has recently attracted much attention due to their high efficacy and low toxicity (23, 34). CA is a phenolic diterpene with anti-cancer effects on hepatocellular carcinoma cells (28, 35) and gastric cancer cells (29). In this study, we showed that CA induced apoptosis in CAL27 and SCC9 cells *in vitro* as well as in the corresponding xenotransplants in BALB/c nude mice through the mitochondrial apoptotic pathway. Moreover, the CA administration to the xenotransplanted mice did not cause any obvious adverse effect on the mice.

As an area for oxidative respiration, the morphology of mitochondria and cristae compartments are closely related to energy metabolism. The critical functions of the mitochondria include cellular energy metabolism, ROS production, mitochondrial Ca^2+^ release, and apoptosis (36). Dysregulation of these functions may lead to the occurrence and development of various diseases, including cancers (37). ROS, by-products of the oxidative metabolism (38), are mainly produced by the electron transport chain (ETC) and play vital roles in apoptosis as well as cell proliferation, differentiation, and metabolism (39). ROS are considered a double-edged sword in tumor cells. Although low levels of ROS may promote cell proliferation, high levels cause oxidative damage, resulting in cell death (37).

Ca^2+^ is a multifunctional second messenger that plays an essential role in the production of ROS (39). Ca^2+^ in the mitochondria promotes the production of ATP and ROS. After Ca^2+^ activation, ROS are directly generated from glycerol phosphate and α-KGDH. Additionally, Ca^2+^ also leads to ROS production by inhibiting complex IV (40–43). Long-term excessive accumulation of mitochondrial Ca^2+^ may trigger mPTP opening, thereby decreasing the MMP and inducing apoptosis (43, 44). Therefore, the balance between ROS and Ca^2+^ levels is essential for mitochondrial functions. CA significantly increased the levels of ROS and CA^2+^ in CAL27 and SCC9 cells, and these increases were accompanied by decreased MMP, destroyed cristae structure of mitochondria and increased apoptosis.

Apoptosis-related disorders can promote the development of cancer (45). Inducing apoptosis in cancer cells by modulating the related signal transduction cascade is one of the strategies to inhibit tumor development (46). Cell-death inducers, such as Ca^2+^ and ROS, activate the endogenous apoptosis (36, 47). In this process, the anti-apoptotic Bcl-2 family proteins are displaced, and the pro-apoptotic proteins Bax and Bad translocate to the mitochondrial outer membrane (OMM) and regulate the mitochondrial outer membrane permeability (MOMP) (48, 49). The formation of Bax and Bad oligomers in the OMM and the excessive accumulation of ROS and Ca^2+^ promote the formation of membrane voids and decrease the MMP (48, 49). Consequently, pro-apoptotic cytokines, including Cyt C, apoptosis-inducing factor (AIF), and second mitochondria-derived activator of caspases (SMAC), are released into the cytoplasm from the mitochondria (50). Cyt C binds to apoptotic protease-activating factor 1 (APAF1) to form a polymer and activates Caspase-9, thereby forming apoptotic bodies. Cleaved Caspase-9 activates Caspase-3, and activation of Caspase-3 is an essential indicator of apoptosis. Activated Caspase-3 cleaves PARP1, which then promotes apoptosis by inducing DNA double-strand breaks (51–54). Flow cytometry and TUNEL staining first proved the pro-apoptotic effect of CA, and then found that CA significantly downregulated Bcl-2 and upregulated Bax, Bad, Cleaved PARP1, and Cleaved caspase-3 and -9 in OSCC cells *in vitro* as well as in the xenotransplant models. Collectively, these results indicate that CA exerts its anti-OSCC effect through the mitochondrial apoptotic pathway.

This study has some limitations. The development of tumors is related to the proliferation of tumor cells and tumor microenvironment. This study only proved that CA induced tumor cells apoptosis, but the regulation between CA and tumor microenvironment still needs to be further explored. In the peripheral blood of the CA-treated mice transplanted with OSCC cells, an increase in the number of monocytes and a decrease in PDWCV were noted. Whether there is anemia or infection in the mice and their relationship with the tumor microenvironment require further analyses.

## Conclusion

This study showed that CA inhibited cell proliferation and migration and induced apoptosis in CAL27 and SCC9 cells by upregulating intracellular ROS and Ca^2+^ and thereby reducing the MMP. Importantly, CA inhibited the tumor growth in BALB/c nude mice transplanted with OSCC cells. The *in vivo* and *in vitro* data presented here indicate that CA suppresses the development of OSCC through the mitochondrial apoptotic pathway. Our findings provide that CA has a valuable pharmacological effect in OSCC cells and OSCC cells-xenotransplanted BALB/c nude mice.

## Data Availability Statement

The original contributions presented in the study are included in the article/**Supplementary Material**. Further inquiries can be directed to the corresponding author.

## Ethics Statement

The animal study was reviewed and approved by Animal Ethics and Welfare Committee of Jilin University.

## Author Contributions

FM and XL contributed equally to this work. MD, YL, and YQ assisted in the experiments. FM and XL prepared the manuscript. WL, FM, and XL revised and drafted the manuscript. WL provided the funding for the study. All authors contributed to the article and approved the submitted version.

## Funding

This work was supported by the International Science and Technology Cooperation Project of Jilin Province Science and Technology Department, China (20200801077GH); Science and Technology Project of Jilin Provincial Department of Finance, China (JCSZ2019378-8); Natural Science Fund Project of Jilin Provincial Science and Technology Department, China (20200201416JC); Changchun Scientfic and Technological Development Program.

## Conflict of Interest

The authors declare that the research was conducted in the absence of any commercial or financial relationships that could be construed as a potential conflict of interest.

## Publisher’s Note

All claims expressed in this article are solely those of the authors and do not necessarily represent those of their affiliated organizations, or those of the publisher, the editors and the reviewers. Any product that may be evaluated in this article, or claim that may be made by its manufacturer, is not guaranteed or endorsed by the publisher.
