# Supplementary material for: Carnosic Acid Suppresses the Development of Oral Squamous Cell Carcinoma via Mitochondrial-Mediated Apoptosis
Source: Front Oncol. 2021 Nov 26;11:760861. doi: 10.3389/fonc.2021.760861 (PMC8662526; doi:10.3389/fonc.2021.760861)
Supplement: Supplementary file 1 [file Table_1.docx]

|  |  | CTRL | CA |
| --- | --- | --- | --- |
|  | Heart | 0.690 ± 0.019 | 0.662 ± 0.088 |
|  | Liver | 6.851 ± 0.098 | 7.084 ± 0.206 |
| CAL27 | Spleen | 0.615 ± 0.063 | 0.654 ± 0.044 |
|  | Lung | 0.719 ± 0.023 | 0.838 ± 0.313 |
|  | Kidney | 1.756 ± 0.029 | 1.857 ± 0.044 |
| SCC9 | Heart | 0.639 ± 0.050 | 0.668 ± 0.097 |
|  | Liver | 6.339 ± 0.665 | 6.367 ± 0.605 |
|  | Spleen | 0.603 ± 0.089 | 0.584 ± 0.061 |
|  | Lung | 0.729 ± 0.086 | 0.768 ± 0.054 |
|  | Kidney | 1.871 ± 0.097 | 1.924 ± 0.169 |

Supplementary Table 1. Effect of CA on organ indexes (%) of CAL27- and SCC9-xenotransplanted nude mice.

Data are expressed as the means ± SD (*n* = 6).

Supplementary Table 2. Effect of CA on peripheral blood of CAL27- and SCC9-xenotransplanted nude mice.

|  |  | CTRL | CA |
| --- | --- | --- | --- |
| CAL27 | WBC (10^9^/L) | 7.317 ± 1.615 | 7.300 ± 0.851 |
|  | LYM (10^9^/L) | 1.720 ± 0.823 | 3.230 ± 0.389 |
|  | LYM% (%) | 26.450 ± 8.709 | 45.417 ± 4.319 |
|  | MON (10^9^/L) | 1.727 ± 0.427 | 1.358 ± 0.299 |
|  | MON% (%) | 22.333 ± 3.397 | 18.267 ± 3.068 |
|  | GRA (10^9^/L) | 3.865 ± 1.388 | 2.708 ± 0.500 |
|  | GRA% (%) | 51.217 ± 9.469 | 36.317 ± 3.540 |
|  | RBC (10^12^/L) | 9.392 ± 0.483 | 8.238 ± 0.598 |
|  | MCV (fL) | 58.450 ± 0.575 | 61.350 ± 2.449 |
|  | HCT (%) | 54.767 ± 2.575 | 50.217 ± 2.317 |
|  | PDWSD (fL) | 28.833 ± 0.307 | 36.000 ± 7.603 |
|  | PDWCV (%) | 12.283 ± 0.101 | 14.183 ± 2.185 |
|  | PLT (10^9^/L) | 346.333 ± 55.422 | 439.000 ± 56.655 |
|  | PCT (%) | 0.351 ± 0.050 | 0.434 ± 0.060 |
|  | MPV (fL) | 10.267 ± 0.263 | 9.767 ± 0.378 |
|  | PDW (fL) | 15.533 ± 1.303 | 13.767 ± 1.335 |
|  | P-LCR (%) | 22.950 ± 2.031 | 20.800 ± 3.583 |
|  | HGB (g/L) | 167.467 ± 8.688 | 156.067 ± 5.574 |
|  | MCH (Pg) | 16.100 ± 1.573 | 19.200 ± 1.613 |
|  | MCHC (g/L) | 305.000 ± 5.416 | 311.333 ± 11.927 |
| SCC9 | WBC (10^9^/L) | 4.417 ± 2.534 | 4.800 ± 1.340 |
|  | LYM (10^9^/L) | 1.000 ± 1.923 | 1.573 ± 1.054 |
|  | LYM% (%) | 15.000 ± 22.107 | 31.333 ± 15.487 |
|  | MON (10^9^/L) | 0.503 ± 0.234 | 0.858 ± 0.373* |
|  | MON% (%) | 12.250 ± 4.060 | 17.583 ± 3.884 |
|  | GRA (10^9^/L) | 2.908 ± 1.476 | 2.362 ± 0.652 |
|  | GRA% (%) | 72.750 ± 21.344 | 51.083 ± 14.452 |
|  | RBC (10^12^/L) | 10.285 ± 0.556 | 9.968 ± 0.492 |
|  | MCV (fL) | 56.800 ± 0.912 | 57.350 ± 0.409 |
|  | HCT (%) | 58.367 ± 3.214 | 57.083 ± 2.564 |
|  | PDWSD (fL) | 29.167 ± 0.753 | 28.833 ± 0.408 |
|  | PDWCV (%) | 12.783 ± 0.172 | 12.533 ± 0.137* |
| SCC9 | PLT (10^9^/L) | 512.000 ± 237.544 | 347 ± 65.195 |
|  | PCT (%) | 0.544 ± 0.216 | 0.381 ± 0.057 |
|  | MPV (fL) | 10.750 ± 0.715 | 10.967 ± 0.543 |
|  | PDW (fL) | 18.367 ± 3.521 | 18.333 ± 2.216 |
|  | P-LCR (%) | 27.917 ± 5.191 | 29.767 ± 4.450 |
|  | HGB (g/L) | 175.083 ± 8.959 | 173.800 ± 8.304 |
|  | MCH (Pg) | 17.000 ± 0.486 | 17.417 ± 0.382 |
|  | MCHC (g/L) | 299.667 ± 9.688 | 304.167 ± 6.047 |

Data are expressed as the means ± SD (*n* = 6). **P* < 0.05 versus control mice.
